# Susceptibility to Seizure-Induced Excitotoxic Cell Death Is Regulated by an Epistatic Interaction between Chr 18 (*Sicd1*) and Chr 15 (*Sicd2*) Loci in Mice

**DOI:** 10.1371/journal.pone.0110515

**Published:** 2014-10-15

**Authors:** Paula Elyse Schauwecker

**Affiliations:** Department of Cell and Neurobiology, USC Keck School of Medicine, Los Angeles, California, United States of America; National Institute of Genetics, Japan

## Abstract

Seizure-induced cell death is believed to be regulated by multiple genetic components in addition to numerous external factors. We previously defined quantitative trait loci that control susceptibility to seizure-induced cell death in FVB/NJ (susceptible) and C57BL/6J (resistant) mice. Two of these quantitative trait loci assigned to chromosomes 18 (*Sicd1*) and 15 (*Sicd2*), control seizure-induced cell death resistance. In this study, through the use of a series of novel congenic strains containing the *Sicd1* and *Sicd2* congenic strains and different combinations of the *Sicd1* or *Sicd2* sub region(s), respectively, we defined these genetic interactions. We generated a double congenic strain, which contains the two C57BL/6J differential segments from chromosome 18 and 15, to determine how these two segments interact with one another. Phenotypic comparison between FVB-like littermates and the double congenic FVB.B6-*Sicd1/Sicd2* strain identified an additive effect with respect to resistance to seizure-induced excitotoxic cell death. It thus appears that C57BL/6J alleles located on chromosomes 18 and 15 interact epistatically in an additive manner to control the extent of seizure-induced excitotoxic cell death. Three interval-specific congenic lines were developed, in which either segments of C57BL/6J Chr 18 or C57BL/6J Chr 15 were introduced in the FVB/NJ genetic background, and progeny were treated with kainate and examined for the extent of seizure-induced cell death. All of the interval-specific congenic lines exhibited reduced cell death in both area CA3 and the dentate hilus, associated with the C57BL/6J phenotype. These experiments demonstrate functional interactions between *Sicd1* and *Sicd2* that improve resistance to seizure-induced excitotoxic cell death, validating the critical role played by gene-gene interactions in excitotoxic cell death.

## Introduction

Epilepsy is a chronic neurologic disorder characterized by the occurrence of spontaneous recurrent seizures, which consist of prolonged and synchronized neuronal discharges. The most common form of epilepsy is temporal lobe epilepsy (TLE), a catastrophic disorder characterized by pharmacologically intractable seizures and progressive cognitive impairment. Hippocampal sclerosis, a pattern of neuronal loss in vulnerable mesial structures of the temporal lobe, is found in about 70% of TLE patients [1], [2], and is characterized by severe segmental neuronal loss in areas CA1, CA3 and the hilar region and is accompanied by pronounced astrogliosis [3]. TLE-associated brain damage is caused by persistent and highly repetitive seizures that are associated with excitotoxic cell death mechanisms. While recent genetic discoveries have led to significant insight into molecular pathways of likely importance in epilepsy pathogenesis [4], these discoveries have not contributed to an understanding of molecular mechanisms that result in seizure-induced cell death.

There is strong evidence that genetic factors are involved in determining individual differences in susceptibility to seizure-induced excitotoxic cell death [5]–[7], but basic research to clarify the role of genetic variants in susceptibility to seizure-induced excitotoxic cell death in the human population is lacking [8]. Because of the genetic heterogeneity of the human population, the genetic dissection of susceptibility to the pathophysiologic sequelae of TLE is very tenuous in human cohorts, and further investigation of the underlying causative alleles and gene interactions are often hindered by genetic heterogeneity, modest gene effect sizes, and complex gene-environment interactions [5], [9], [10]. The use of inbred mouse strains provides a more tractable approach for investigating disease loci.

Although there are no known inbred strains that spontaneously develop status, researchers have used induced models of status in experimental animals such as mice. Many of the pathophysiological consequences of human TLE (e.g. hippocampal sclerosis, mossy fiber sprouting, spontaneous seizures) are faithfully reproduced in the kainic acid (KA) chemoconvulsant rodent model of epilepsy [11]–[14]. Kainic acid, a potent agonist of the α-amino-3-hydroxy-5-methyl-4-isoxazoleproprionic acid/kainate class of glutamate receptors, is a powerful excitant and excitotoxin, which when injected directly into the brain or systemically induces a characterized pattern of persistent seizure activity [15] and selectively induces excitotoxic cell death in postsynaptic neurons in the CA3 and CA1 hippocampal subfields and within the dentate hilus [16]–[19]. Thus, KA administration has been widely used as a model to study excitotoxicity and seizure related neurologic diseases [15], [20]. Among mouse models of epilepsy, genetic background is known to affect both seizure susceptibility to chemoconvulsants [21]–[25], as well as susceptibility to the neuropathological consequences of seizures [26]–[31].

Previous studies in our lab as well as others have shown that the genetic background of mice significantly affects the susceptibility of hippocampal neurons to damage by systemic kainate administration [26], [27], [29], [30]. We have identified two strains of mice, C57BL/6J (B6) and FVB/NJ (FVB), which differ in both their genotype and exhibit a maximum difference in susceptibility to seizure-induced excitotoxic cell death [29]. To identify genes involved in susceptibility to seizure-induced excitotoxic cell death, we have taken a genetic approach [30] to analyze these inbred mouse strains. We previously analyzed seizure-induced excitotoxic cell death susceptibility in a backcross generated between the susceptible FVB/NJ strain and the resistant C57BL/6J strain, and reported that the robust difference in seizure-induced excitotoxic cell death susceptibility between these two strains is a multifactorial trait determined by three genomic regions on mouse chromosomes (Chrs) 4, 15, and 18. On both Chr 18 and 15, B6 alleles were associated with reduced susceptibility to seizure-induced excitotoxic cell death, whereas FVB alleles are associated with increased susceptibility. We designated these loci as *Sicd*, for seizure-induced cell death.

In previous studies, we isolated congenic strains containing either the relevant B6-derived Chr 18 interval in the susceptible FVB mouse (*Sicd1*; [32]), or the relevant B6-derived Chr 15 interval in the susceptible FVB mouse (*Sicd2*; [33]) and confirmed both QTL. Both of these congenic strains showed reduced susceptibility to seizure-induced excitotoxic cell death, providing evidence that both of these loci examined contribute to reduced susceptibility to seizure-induced excitotoxic cell death. In theory, combining both of these *Sicd* loci in the susceptible (FVB) genetic background should allow greater protection against seizure-induced excitotoxic cell death. Thus, we were interested in verifying the genetic interaction between the two loci through the creation of a double congenic line, *Sicd1* + *Sicd2*. We have constructed such a double congenic strain to determine whether these two QTLs affect the susceptibility phenotype in an additive manner.

In the present study, we generated a double congenic strain harboring both *Sicd1* and *Sicd2* derived from C57BL/6J (B6) mice, and also developed a series of novel double congenic mice isolating *Sicd1* and *Sicd2* sub regions and recombining the isolated regions back together to create various recombinations, and investigated their susceptibility to seizure-induced excitotoxic cell death. Our findings suggest that while B6-derived alleles for *Sicd1* or *Sicd2* alone can reduce susceptibility to seizure-induced excitotoxic cell death when bred into the FVB genetic background, the combination of B6-derived alleles at *Sicd1* and *Sicd2* in the FVB genetic background is able to confer increased resistance to seizure-induced excitotoxic cell death.

## Materials and Methods

### Ethics statement

Animal care and use were carried out in strict accordance with the recommendations in the Guide for the Care and Use of Laboratory Animals of the National Institutes of Health. All procedures were approved by the University of Southern California Institutional Animal Care and Use Committee under protocol #11638.

### Animals and generation of congenic strains

**Figure 1:**
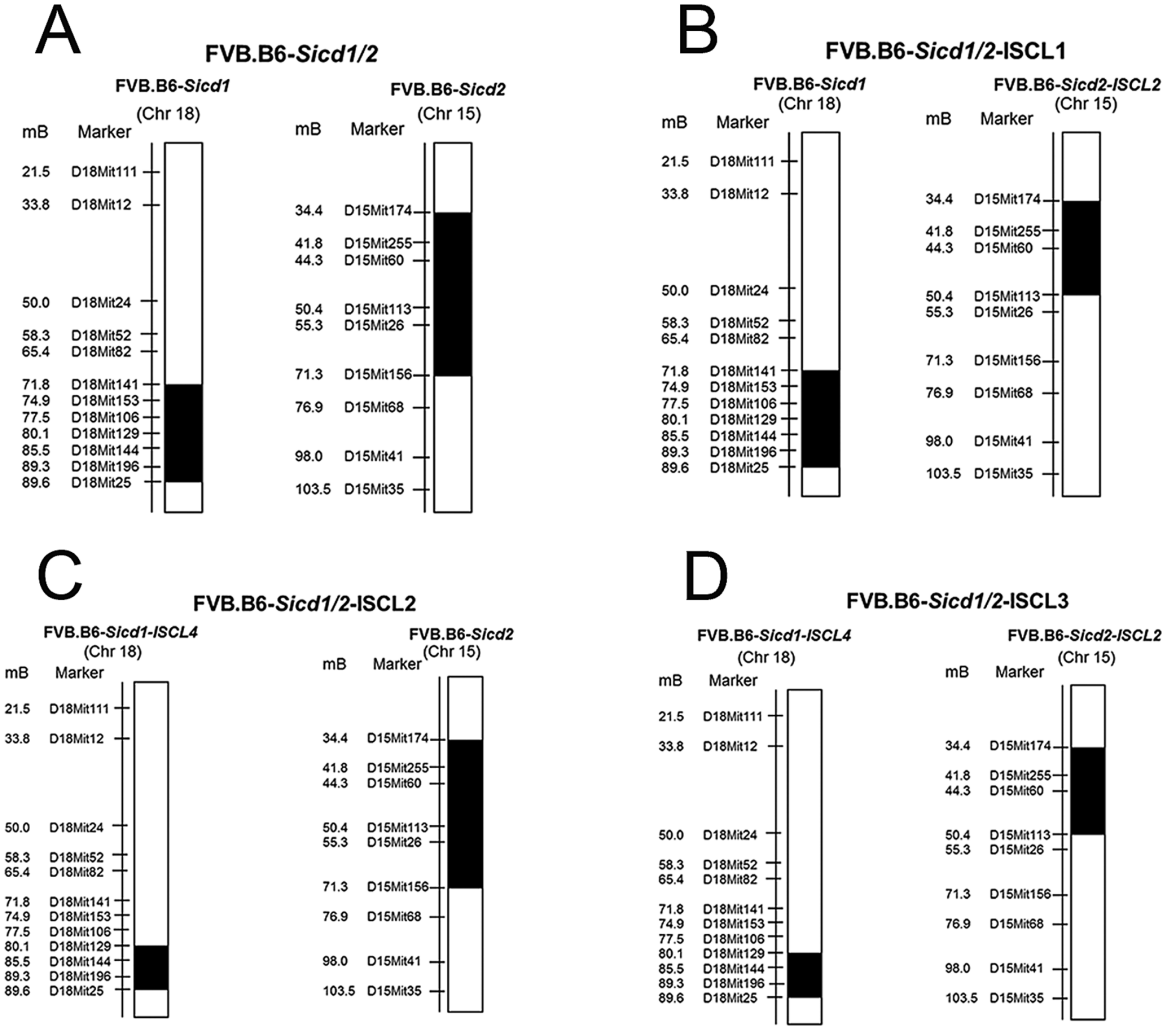
Genetic maps of the Chrs 18 and 15 QTL regions introgressed in the congenic strains. Schematic representation of the 4 congenic strains (**A**: FVB.B6-*Sicd1/Sicd2*, **B**: FVB.B6-*Sicd1/Sicd2*-ISCL1, **C**: FVB.B6-*Sicd1/Sicd2*-ISCL2, and **D**: FVB.B6-*Sicd1/Sicd2*-ISCL3) in which segments from the B6 parent strain were introgressed in the FVB genetic background. Strains were produced as described in Materials and Methods. The names of DNA microsatellite markers typed for these studies and used for selecting interval-specific recombinants are indicated on the left for each given chromosome. Their relative positions (in Mbp from the acromere) on the physical map (http://genome.ucsc.edu) are given. Black regions reflect contributions from the B6 parental strain, while white regions reflect contributions from the FVB parental strain.

The animals used in these studies were derived from C57BL/6J (B6) and FVB/NJ (FVB) mice originally purchased from Jackson Laboratory (Bar Harbor, ME) and propagated at the Zilkha Neurogenetic Institute at the USC Keck School of Medicine. Genetic drift in the colony is minimized by supplementing breeders with additional animals purchased from the Jackson Laboratory several times per year. Single congenic (FVB.B6-*Sicd1*, FVB.B6-*Sicd2*), interval-specific congenic strains of FVB.B6-*Sicd1*-ISCL4 and of FVB.B6-*Sicd2*-ISCL2, and double congenic (FVB.B6-*Sicd1/Sicd2*), and FVB-like littermate controls were used. The FVB.B6-*Sicd1* and FVB.B6-*Sicd2* congenic mice used in these studies were created in our laboratory using a marker-assisted speed congenic strategy as described previously [32], [33]. For the *Sicd1* single congenic strain, the region is on Chr 18 between markers D18Mit141 (71.8 Mb) and D18Mit25 (89.6 Mb), spanning 17.8 Mb (see Figure 1), about 20% of the chromosome. In the *Sicd2* single congenic strain, the region is on Chr 15 between markers D15Mit174 (33.9 Mb) and D15Mit156 (71.15 Mb), spanning 37.3 Mb (see Figure 1), about 36% of the chromosome.

The double congenic strain containing the *Sicd1* (Chr 18) and *Sicd2* (Chr 15) QTLs, was generated by intercrossing mice congenic for each of the two differential segments [32], [33]. Animals from the resultant F1 progeny were intercrossed and F2 mice were screened for B6 homozygosity at *Sicd1* and *Sicd2*. The established strain, designated FVB.B6-*Sicd1/Sicd2*, was subsequently maintained by brother-sister mating.

The FVB.B6-*Sicd1*-ISCL4 and FVB.B6-*Sicd2*-ISCL2 sub-congenic strains used for breeding of FVB.B6-*Sicd1/Sicd2*-ISCL1, FVB.B6-*Sicd1/Sicd2*-ISCL2 and FVB.B6-*Sicd1/Sicd2*-ISCL3 were developed and bred in our colony at the Zilkha Neurogenetic Institute at the University of Southern California Keck School of Medicine as previously described [32], [33]. Briefly, individual congenic recombinant mice (FVB.B6-*Sicd1* or FVB.B6-*Sicd2*) were backcrossed to FVB mice, resulting in multiple offspring with the same recombination (also referred to as an interval specific congenic line, ISCL). Individual progeny were genotyped using four and 12 microsatellite markers within or flanking the seizure-induced cell death susceptibility QTL on mouse Chr 18 (*Sicd1*) or Chr 15 (*Sicd2*) to identify recombinant mice and define the boundaries of the introgressed region. The recombinant mice used to generate the ISCLs carried a unique recombination event somewhere in the introgressed region. These mice were heterozygous for a reduced B6 interval; they were then intercrossed to produce homozygotes, which were intermated to produce and maintain the ISCLs. A final intercross using ISCL animals was performed to isolate the donor homozygotes, which constituted the finished ISCL strain. No genotyping, other than on the B6 interval, was done in the ISCLs as the backcross was always to FVB. The three double congenic strains: FVB.B6-*Sicd1/Sicd2*-ISCL1, FVB.B6-*Sicd1/Sicd2*-ISCL2 and FVB.B6-*Sicd1/Sicd2*-ISCL3 were generated by intercrossing mice congenic for each of the two respective differential segments (see Figure 1). Animals from the resultant F1 progeny were intercrossed and F2 mice were screened for B6 homozygosity at *Sicd1* and *Sicd2*-ISCL2 (for *Sicd1/Sicd2*-ISCL1), *Sicd1*-ISCL4 and *Sicd2* (for *Sicd1/Sicd2*-ISCL2), and *Sicd1*-ISCL4 and *Sicd2*-ISCL2 (for *Sicd1/Sicd2*-ISCL3), respectively. The established strains, designated FVB.B6-*Sicd1/Sicd2*-ISCL1, FVB.B6-*Sicd1/Sicd2*-ISCL2, and FVB.B6-*Sicd1/Sicd2*-ISCL3, were subsequently maintained by brother-sister mating.

### DNA extraction and microsatellite genotyping

High molecular mass mouse tail DNA was used as a template for polymerase chain reaction (PCRs), and genomic DNA was extracted from the tail of the mouse according to a previously published protocol [34]. Briefly, a small piece (∼5 mm) of the tip of the tail was cut off with sharp scissors and placed into an Eppendorf tube. Tail tips were incubated for 1 hour at 98°C in 75 µl of lysis solution containing 25 mM NaOH and 0.2 mM EDTA. The temperature was then reduced to 15°C and 75 µl of 40 mM Tris-HCl (pH 5.5) was added to the tube. Tubes were then centrifuged at 4000 rpm for 3 minutes and DNA was isolated using standard procedures for ethanol precipitation, resuspended in Tris-EDTA (10 mM Tris, 1 mM Na_2_-EDTA, pH 7.4), and stored at 4°C. Concentrations of DNA were determined spectrophotometrically, and samples diluted to 100 ng/µl.

Microsatellite genotyping was conducted as previously described [30]. In brief, the procedure was as follows. Microsatellite primers were purchased from Sigma Aldrich (St. Louis, MO) and were selected on the basis of their map locations and on their being polymorphic between parental strains (http://www.genome.mit.edu). After a 5 min incubation period at 94°C, the reactions were amplified through 35 cycles of 25 s at 94°C, 25 s at 58°C and 30 s at 72°C, followed by 5 min at 72°C. For markers with allele differences of ≥8 bp, we used a non-isotopic method of analysis involving sizing of the PCR products by loading them onto 4% agarose gels (Genepure Hi Res Agarose; ISC Bioexpress, Kaysville, UT) and visualizing them with GelRed nucleic acid gel staining (Biotium, Hayward, CA) of PCR products. After gel electrophoresis, two independent scorers recorded genotypes from gels analyzed with a digital gel documentation system (Gel-DocIt; UVP, Upland, CA) that provides digital images from nucleic acid-stained gels. To minimize genotyping errors, any discrepancies or inconsistencies in genotype readings were resolved by either retyping or discarding questionable genotypes. The map positions of the microsatellite markers and genes were derived from either the April 2013 (NCBI Build m38) release of the Mouse Genome Sequencing Consortium (http://www.genome.ucsc.edu/) or the megabase position from the Mouse Genome Database (http://www.informatics.jax.org/June 2013).

### Systemic kainic acid (KA) administration and behavioral assessment of seizures

Young adult mice (6–8 weeks old)(FVB/NJ, FVB.B6-*Sicd1*, FVB.B6-*Sicd2*, FVB.B6-*Sicd1/Sicd2*, FVB.B6-*Sicd1/Sicd2*-ISCL1, FVB.B6-*Sicd1/Sicd2*-ISCL2, and FVB.B6-*Sicd1/Sicd2*-ISCL3) were used in these studies. Sustained seizures (SE) were induced in animals by the administration of kainic acid (KA), a potent agonist of the AMPA/KA class of glutamate receptors. KA (A.G. Scientific, San Diego, CA) was dissolved in isotonic saline (pH 7.3) and administered subcutaneously to adult mice at a dose of 25 mg/kg. To minimize mortality because of dehydration or starvation, animals were fed moist high-fat rodent chow until animals were observed to be eating dry chow. In our experience, 20% of mice die during SE.

Mice were monitored, behaviorally by an observer, continuously for 4 h for the onset and extent of seizure activity. The behavioral progression of KA-induced seizures was rated and recorded using a previously defined six-point seizure scoring scale [29] that was adapted from a five-point scale for rats [35]. The behavioral progression of KA-induced seizures was rated and recorded using the following classification: stage 1, immobility; stage 2, forelimb and/or tail extension, rigid posture; stage 3, repetitive movements and head bobbing; stage 4, rearing and falling; stage 5, continuous rearing and falling; and stage 6, severe tonic-clonic seizures. SE was defined as continuous behavioral seizure activity that lasted for at least 30 min or a series of intermittent seizures without restoration of normal behavioral patterns between successive seizures. Only those mice exhibiting at least 45 min of continuous stage 4/5 seizures were included in the study, as previous studies have suggested that there is a direct relationship between the generation of epileptiform activity and the extent of damage in hippocampal subfields [16], [36],[37]. Seizure parameters monitored included latency of convulsions and duration of severe (stage 4/5) seizure activity. All experiments were approved by the Institutional Animal Care and Use Committee of the University of Southern California, and conducted in accordance with its guidelines.

### Tissue preparation and histological staining

To evaluate the severity of brain damage associated with seizures, brains from mice of each genotype were processed for cresyl violet staining to assess cell loss and morphology, according to previously published methods [30], [32]. Briefly, 7 days following KA administration, mice were anesthetized and transcardially perfused with 4% paraformaldehyde in 0.1 M phosphate buffer (pH 7.4). Brains were removed and post-fixed overnight. Brains were left in 30% sucrose for at least 12–18 h for cryoprotection. Horizontal (40 µm) frozen sections were cut on a sliding microtome, and immersed in 0.1 M phosphate buffer (pH 7.4), free-floating, until histological processing had begun. Every sixth section (∼240 µm) was processed for cresyl violet staining to assess cell loss and morphology.

Additional sections were stained with Fluoro-Jade, a fluorescent marker for dying neurons, according to a method outlined previously [38]. Briefly, horizontal sections were mounted onto gelatin-coated slides and allowed to air-dry. Slides were then immersed in absolute alcohol for 3 min, followed by 70% ethanol for 1 min, and distilled water for 1 min. The slides were then transferred to 0.06% potassium permanganate for 15 min. After rinsing with distilled water for 1 min, the slides were then incubated for 30 min in 0.001% Fluoro-Jade C solution (EMD Millipore, Temecula, CA) made in 0.1% acetic acid. Slides were rinsed briefly in water, allowed to air-dry and coverslipped with non-fluorescent mounting media (Kirkegaard and Perry Laboratories, Gaithersburg, MD). Tissue sections were examined with an epifluorescent microscope equipped for visualizing fluorescein isothiocyanate.

### Morphological assessment of neuronal damage

To provide an overall view of genotype-dependent effects on cell loss throughout the hippocampus, neuronal degeneration was evaluated in sections stained with cresyl violet. The number of degenerating neurons in both the right and left hippocampus from every sixth section (∼240 µm separation distance) in CA3, CA1, dentate hilus and dentate gyrus was estimated and a histological damage score was assigned on a 0–3 grading scale according to the following criteria: grade 0, absence of pyknotic cells; grade 1.0, mild (<25% of cell pyknotic); grade 2.0, moderate (<50% of hippocampal neurons pyknotic); and grade 3.0, extensive (>50% of cells pyknotic) according to a previously defined scale [30], [39]–[42]. All grading was performed by an observer blinded to the treatment groups.

Values for right and left hemispheres were averaged for each mouse. For the hippocampus, scores from sections were averaged and used for calculating group values. As histological damage scores were normally distributed, we were able to use standard parametric methods of data analysis. Thus, to determine whether differences in histological scores existed among the groups of mice, results were assessed statistically by one-way analysis of variance (ANOVA) with the computer program SigmaStat (Jandel Scientific, San Rafael, CA) and intergroup differences were analyzed by Student Newman-Keuls post-hoc test.

### Quantitative analysis of hippocampal cell loss

To determine the susceptibility of individual hippocampal subfields to neurotoxic insult, we counted neurons in Nissl-stained sections. Quantitative analysis of hippocampal cell loss was performed by an observer blinded to the groups using unbiased stereological methods on cresyl violet-stained sections according to previously published protocols [29], [43], [44]. The number of Nissl-stained neurons was counted in both right and left hemispheres in area CA3, area CA1, the dentate hilus, and the dentate gyrus. Hippocampal subfields were based on Franklin and Paxinos classification [45], and discrimination between the CA3 and dentate hilus region was based on morphological features and locations of the cells [46]. Specifically, for dentate hilar cell counts, the hilus was operationally defined as the region bordered by the supra- and infrapyramidal granule cell layers and excluding the densely packed pyramidal neurons of area CA3.

**Figure 2:**
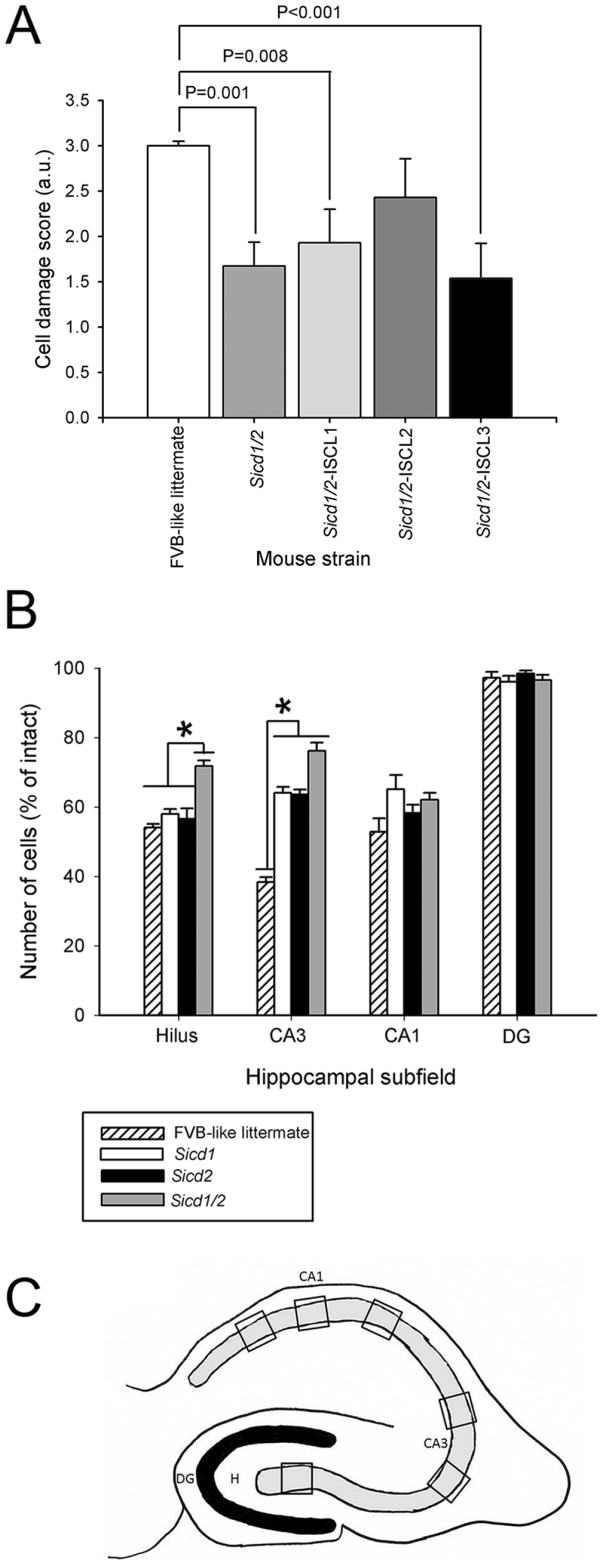
Confirmation that the FVB.B6-*Sicd1/Sicd2* double congenic strain contains gene(s) that influence susceptibility to seizure-induced cell death. A. Data represent neuronal damage scores for the entire right and left hippocampus [in arbitrary units (a.u.), mean ± standard error of the mean (SEM)] for FVB.B6-*Sicd1*, FVB.B6-*Sicd2*, FVB.B6-*Sicd1/Sicd2*, and FVB-like littermates. The extent of neuronal damage was indexed with a histological damage score. A statistically significant difference in cell loss throughout the hippocampus was observed in FVB.B6-*Sicd1* single congenics (F_1,56_ = 8.89, P = 0.004), FVB.B6-*Sicd2* single congenics (F_1,34_ = 7.30, P = 0.011), and FVB.B6-*Sicd1/Sicd2* double congenics (F_1,43_ = 11.75, P = 0.001) at 7 days following KA administration as compared to FVB-like littermates. B. Quantitative analysis of neuronal density in hippocampal subfields following kainic acid (KA) administration to FVB.B6-*Sicd1* single congenic, FVB.B6-*Sicd2* single congenic, FVB.B6-*Sicd1/Sicd2* double congenic, and FVB-like littermate mice. A strain-dependent difference in cell loss in the dentate hilus (F_3,111_ = 8.57; P<0.001) and area CA3 (F_3,111_ = 20.68; P<0.001) was observed at 7 days following KA administration. Data represent the mean ± SEM of at least 14 mice/strain. C. The hippocampal cartoon shown illustrates the sampling strategy adopted for cell counts, based on six window frames placed throughout the pyramidal layers. CA1 and CA3, hippocampal subfields; DG, dentate gyrus.

Neuron counts were made in all subfields and in both hemispheres of each mouse. Values for each side were averaged into single values for each animal. Surviving cells were counted only if they were contained within the pyramidal cell layer and dentate hilus, possessed a visible nucleus and characteristic neuronal morphology, and had a cell body larger than 10 µm. Counting was initiated within the ventral hippocampus at the first point where hippocampal subfields could be easily identified. This level corresponded to horizontal section 54, based on the atlas of Sidman et al. [47]. Six square counting frames (200×200 µm) were randomly placed in the pyramidal layer of fields CA1 and CA3 or in the dentate hilus in four to five regularly spaced horizontal sections from each animal that were at least 240 µm apart (Figure 2C). Only those neuronal nuclei in the focal plane were counted with a 40X objective and considered as a counting unit. Neuronal counting was performed with the aid of Image Pro-Plus software (Media Cybernetics, Inc., Silver Spring, MD) in combination with a SPOT digital camera (Diagnostic Instruments, Inc., Sterling Heights, MI) and a motorized Z-stage (Optiscan, Prior Scientific, Fairfax, VA). All data were expressed as average number of neurons per field, and final cell counts were expressed as the percentage of cells as compared with intact mice.

### Statistical analysis

SigmaStat (Jandel Scientific, San Rafael, CA) was used for all statistical tests. Data were presented as mean ± SEM, and differences between groups were compared statistically by one-way ANOVA. This comparison was used to determine the mean difference in susceptibility expected from the introgressed B6 or FVB, respectively, region and the 95% confidence interval for that difference. One-way ANOVA with Student Newman-Keuls post hoc multiple comparison test was used to assess all double congenic and double congenic ISCLs for significant reduction in the extent of cell damage relative to FVB-like littermates. Mean values are given for phenotypes ± SEM. Data were considered to be statistically significant when P<0.05.

## Results

### Construction of the double congenic strain (FVB.B6-*Sicd1/Sicd2*)

Previous QTL analysis [30] identified a *Sicd1* locus on mouse Chr 18 and a *Sicd2* locus on mouse Chr 15 that regulated susceptibility to seizure-induced cell death. Both QTLs have been confirmed in congenic mice using congenic strains that carry resistant alleles at the refined QTL on Chr 18 [32] or Chr 15 [33] from the resistant B6 strain on a susceptible background (FVB). To examine if and how these loci cooperate in the context of seizure-induced cell death, a double congenic strain, FVB.B6-*Sicd1/Sicd2*, was generated. We constructed a double congenic mouse strain characterized by a segment from mouse B6 Chr 18 (from D18Mit141 to D18Mit25) and a segment from mouse B6 Chr 15 (from D15Mit174 to D15Mit156), containing the QTL intervals originally defined as controlling seizure-induced cell death, introgressed into the FVB background. The extent of the introgressed regions for the FVB.B6-*Sicd1/Sicd2* congenic line is shown in Figure 1A. We selected for animals non-recombinant for the detected QTL intervals, as defined by the flanking markers. Polymorphic markers outside the target region were genotyped to confirm the absence of B6 genomic components. The final congenic line was homozygous for the selected regions in all cases.

### Seizure effects following systemic administration of KA to FVB.B6-*Sicd1/Sicd2* mice

**Figure 3:**
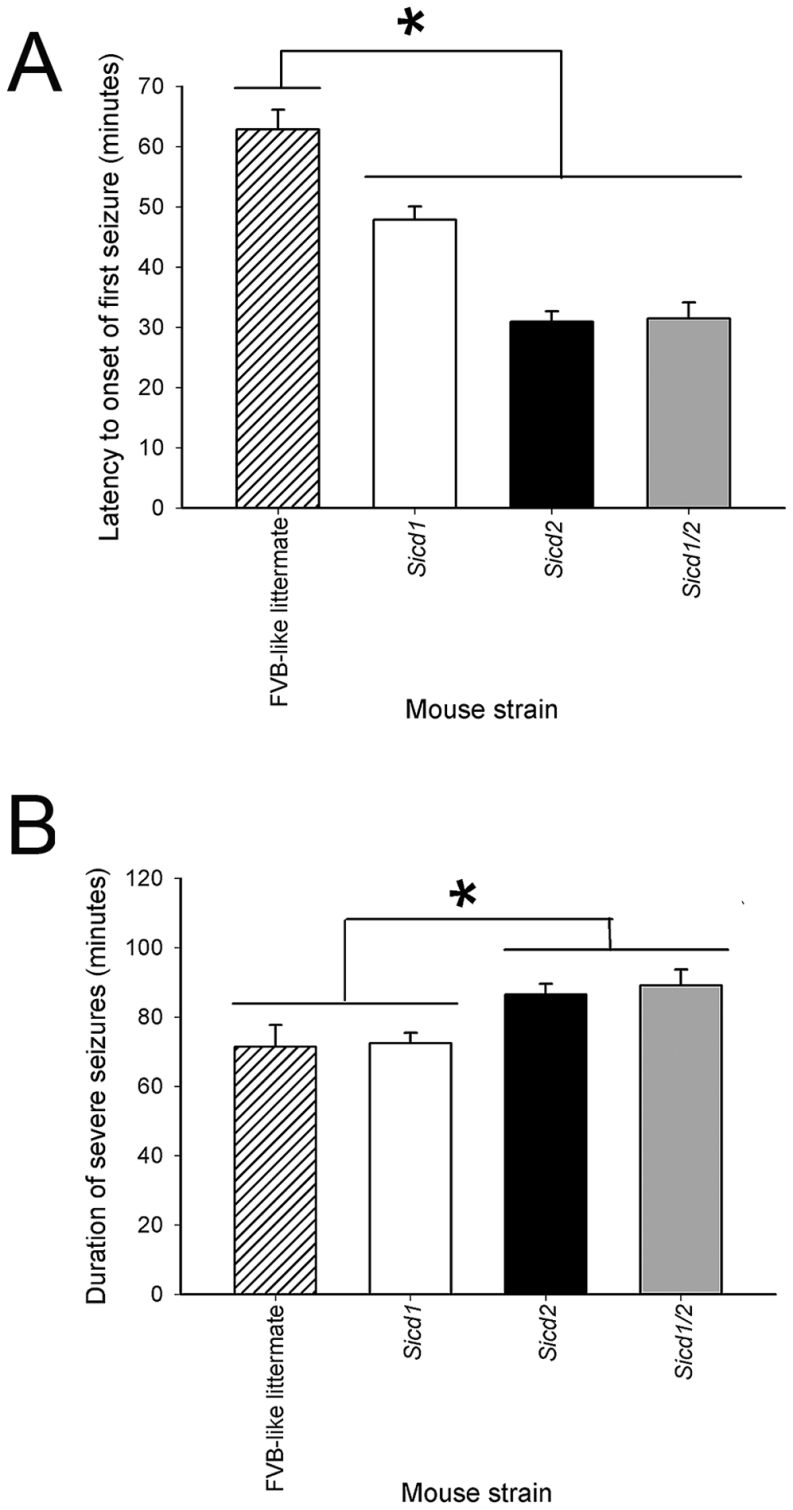
Histograms of seizure parameters in FVB.B6-*Sicd1*, FVB.B6-*Sicd2*, and FVB.B6-*Sicd1/Sicd2* mice following KA-induced status epilepticus. A. Data represent latency scores in minutes (mean ± SEM) for FVB.B6-*Sicd1* (n = 45), FVB.B6-*Sicd2* (n = 45), FVB.B6-*Sicd1/Sicd2* (n = 30), and FVB-like littermate (n = 14) strains. ANOVA testing showed a significant reduction in latency (F_3,133_ = 29.43; P<0.001) between all congenic strains and FVB-like littermates. B. Data represent the seizure duration scores in minutes (mean ± SEM) for FVB.B6-*Sicd1*, FVB.B6-*Sicd2*, FVB.B6-*Sicd1/Sicd2*, and FVB-like littermate strains. ANOVA testing showed a significant increase in duration (F_2,88_ = 3.16; P = 0.04) of severe seizures between FVB.B6-*Sicd2*, FVB.B6-*Sicd1/Sicd2*, and FVB-like littermates.

Kainic acid (KA) is a potent neurotoxin that, when injected systemically, produces epileptic behavior and subsequent neurodegeneration [36], [48]–[50]. To determine if seizure severity was modulated in a double congenic strain, FVB.B6-*Sicd1/Sicd2* mice were treated with kainic acid and we examined whether significant differences in latency to the onset of the first severe seizure or duration of severe seizures differed significantly from the single congenic strains *Sicd1* and *Sicd2*. As previously reported [20], [51]–[53], administration of KA causes characteristic sequential behavioral changes. Within 15 minutes after a systemic injection of KA into *Sicd1*, *Sicd2* and *Sicd1/Sicd2* mice, all animals began to exhibit behavioral signs of convulsive seizures as previously reported [29], [30], [44]. Within 30–45 min after injection, all mice exhibited continuous tonic-clonic seizures that lasted for 1–2 hr. As shown in Figure 3, *Sicd1/Sicd2* congenic mice exhibited a latency to the first severe seizure that was not statistically different from *Sicd2* congenic mice, but was significantly reduced as compared to FVB-like littermate mice (F_1,43_ = 49.29; P<0.001). In particular, a reduction by nearly 50% was observed in *Sicd1/Sicd2* with regard to shortened latency to onset of severe seizures, as compared with FVB-like littermates. Similarly, the duration of severe (stage 4/5) seizures was significantly increased in *Sicd1/Sicd2* mice as compared to FVB-like littermates (F_1,43_ = 4.87; P = 0.033) or *Sicd1* congenic mice. Saline-treated mice of all genotypes showed no signs of epileptic activity.

### Two seizure-induced cell death loci contribute to resistance to seizure-induced cell death

Consistent with previous studies in mice [29], [30], [32], [33], administration of KA to FVB-like littermates or FVB.B6-*Sicd1* or FVB.B6-*Sicd2* congenic mice led to the degeneration and loss of CA3 pyramidal neurons and hilar neurons and sporadic loss of CA1 pyramidal neurons. In accordance with previous studies [37], [54]–[56], neurons within the dentate granule cell layer and area CA2 of Ammon's horn were spared. Phenotypic results for the 3 congenic strains that underwent kainate testing are shown in Figure 2. Susceptibility to seizure-induced cell death was analyzed as a quantitative trait by using a semiquantitative histological scoring system, as previously described [30]. All mice could be assigned to distinct phenotypic classes and were scored as having evidence of no degeneration (grade 0), mild degeneration (grade 1), moderate degeneration (grade 2), or extensive degeneration (grade 3). As previously shown [32], [33], histopathological evaluation confirmed that FVB.B6-*Sicd1* and FVB.B6-*Sicd2* mice exhibited significantly reduced susceptibility to seizure-induced cell death compared to FVB-like littermates (Figure 2A). Cell damage scores throughout the whole hippocampus for FVB.B6-*Sicd1* mice averaged 1.89±0.21, while FVB.B6-*Sicd2* mice averaged 2.11±0.27, as compared to 3.00±0.05 for FVB-like littermates. In contrast, FVB.B6-*Sicd1/Sicd2* double congenic mice exhibited significantly reduced neuronal damage (1.68±0.26, in arbitrary units) than did the FVB-like littermates (3.00±0.05, in arbitrary units).

**Figure 4:**
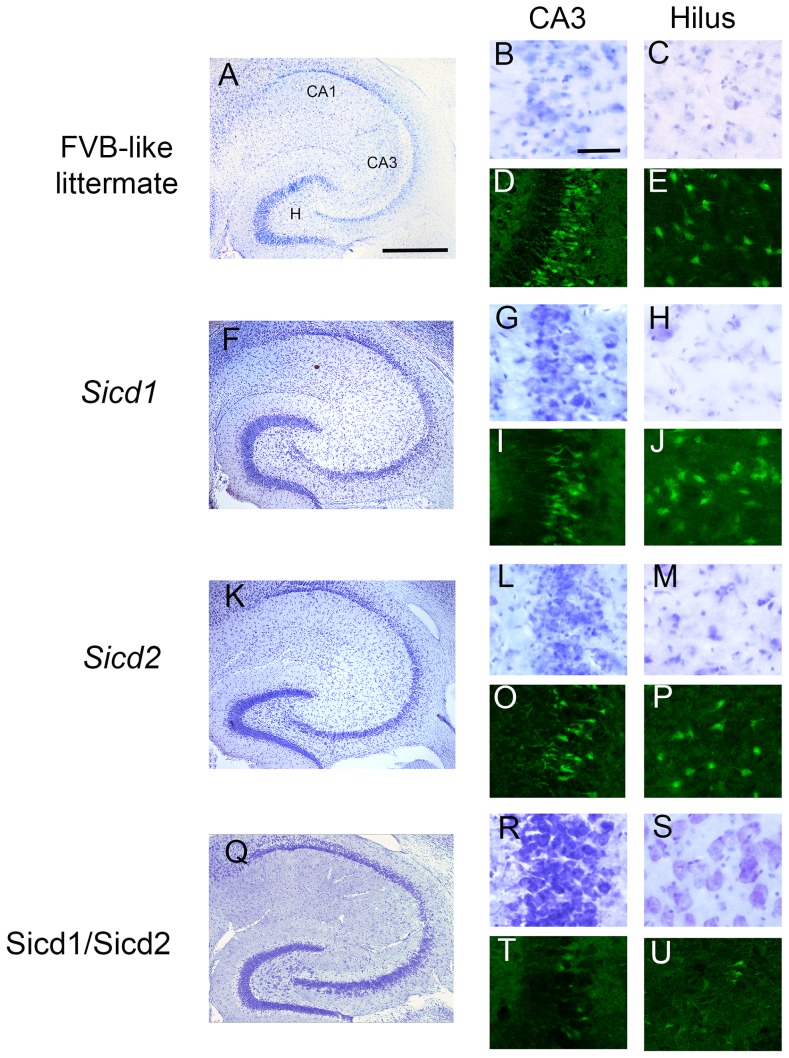
Kainate-induced cell death susceptibility is reduced in the FVB.B6-*Sicd1/Sicd2* double congenic strain. Susceptibility to kainate-induced cell death in FVB-like littermates, FVB.B6-*Sicd1* congenic strain, FVB.B6-*Sicd2* congenic strain, and FVB.B6-*Sicd1/Sicd2* double congenic strain at 7 days following systemic administration of kainic acid. Low-magnification photomicrographs of cresyl violet-stained horizontal sections of the hippocampus illustrating surviving cells throughout the hippocampus (A,F,K, and Q). High-magnification cresyl violet staining of area CA3 (B, G, L, andR) and the dentate hilus (C, H, M, and S) in FVB-like littermates (B,C), FVB.B6-*Sicd1* (G,H), FVB.B6-*Sicd2* (L,M), and FVB.B6-*Sicd1/Sicd2* (R,S) and high-magnification Fluoro-Jade staining of area CA3 and the dentate hilus in FVB (D,E), FVB.B6-*Sicd1* (I,J), FVB.B6-*Sicd2* (O,P), and FVB.B6-*Sicd1/Sicd2* (T,U). Note that a reduction in the extent of cell death is observed throughout area CA3 in all congenic strains (G,I,L,O,R,T) as compared to FVB-like littermates (B,D). A dramatic reduction in the extent of cell death was observed in the dentate hilus only in FVB.B6-*Sicd1/Sicd2* double congenic mice (S,U) as compared to FVB-like littermates (C,E). CA1 and CA3, hippocampal subfields; H, dentate hilus. Scale bars: 750 µm (A,F,K, and Q) and 100 µm (B,C,D,E,G,H,I,J,L,M,O,P,R,S,T, and U).

To evaluate cell loss and neurodegeneration, sections from each of the congenic strains (FVB.B6-*Sicd1*, FVB.B6-*Sicd2*, and FVB.B6-*Sicd1/Sicd2*), killed 7 days following kainate injection and processed for cresyl violet and Fluoro-Jade staining, are shown in Figure 4. Cresyl violet, which reveals Nissl substance (RNA) within intact neuronal cell bodies and Fluoro-Jade B, a specific fluorescent marker that is a reliable indicator of degenerating neurons as compared to cresyl violet, were used to evaluate cell loss and neurodegeneration 7 days after kainate administration. As seen in Figure 4, all congenic strains showed a reduction in the extent of cell death within area CA3, as compared to FVB-like littermates (FVB.B6-*Sicd1*:G,I; FVB.B6-*Sicd2*: L,O; and FVB.B6-*Sicd1/Sicd2*: R,T). However, FVB.B6-*Sicd1/Sicd2* double congenic mice showed a more significant reduction in the extent of cell death in area CA3 as shown in Figure 4 (Q,R,T). Moreover, only FVB.B6-*Sicd1/Sicd2* double congenic mice showed a dramatic reduction in cell death throughout the dentate hilus as well (Fig. 4S,U), as compared to either FVB-like littermates (Fig. 4C,E), FVB.B6-*Sicd1* congenic mice (Fig. 4H,J), or FVB.B6-*Sicd2* congenic mice (Fig. 4M,P).

As shown in Figure 2B, quantitative analysis of hippocampal subfield group means showed that all congenic strains exhibited a significant protective effect in area CA3 as compared with the FVB-like littermates (F_3,111_ = 20.68; P<0.001). However, the addition of the FVB.B6-*Sicd2* locus to the FVB.B6-*Sicd1* genetic background resulted in a statistically significant increase in resistance to seizure-induced cell death in area CA3 over those of either of the two single congenic strains (FVB.B6-*Sicd1* or FVB.B6-*Sicd2*). In particular, FVB.B6-*Sicd1/Sicd2* double congenic mice lost on average 35% fewer CA3 neurons than FVB-like littermates and 12% fewer CA3 neurons than either FVB.B6-*Sicd1* or FVB.B6-*Sicd2*. Within the dentate hilus, only FVB.B6-*Sicd1/Sicd2* displayed a statistically significant reduction (28% cell loss) in cell death as compared with FVB-like littermates and FVB.B6-*Sicd1* or FVB.B6-*Sicd2* congenic mice (F_3,111_ = 8.569; P<0.001). Within the CA1 subfield, no significant reduction in the extent of cell death was observed among any of the congenic strains.

### Higher-resolution mapping of *Sicd1/Sicd2*

We generated three ISCLs for further refinement of the *Sicd1/Sicd2* loci. ISCL strains were detected by screening for recombination between the flanking markers used to construct the congenic lines. FVB.B6-*Sicd1/Sicd2*-ISCL1 was validated as carrying a unique recombination event and derived from the original FVB.B6-*Sicd2* congenic strain (Figure 1B). FVB.B6-*Sicd1/Sicd2*-ISCL2 was validated as carrying a unique recombination event and derived from the original FVB.B6-*Sicd1* congenic strain (Figure 1C). Figures 1B, C, and D show the overlapping genetic markers used to define and characterize these three interval-specific congenic strains together with their mB positions. Genetic markers throughout the B6 donor region were characterized in each ISCL.

### Kainate-induced seizure severity in interval-specific double congenic lines

**Figure 5:**
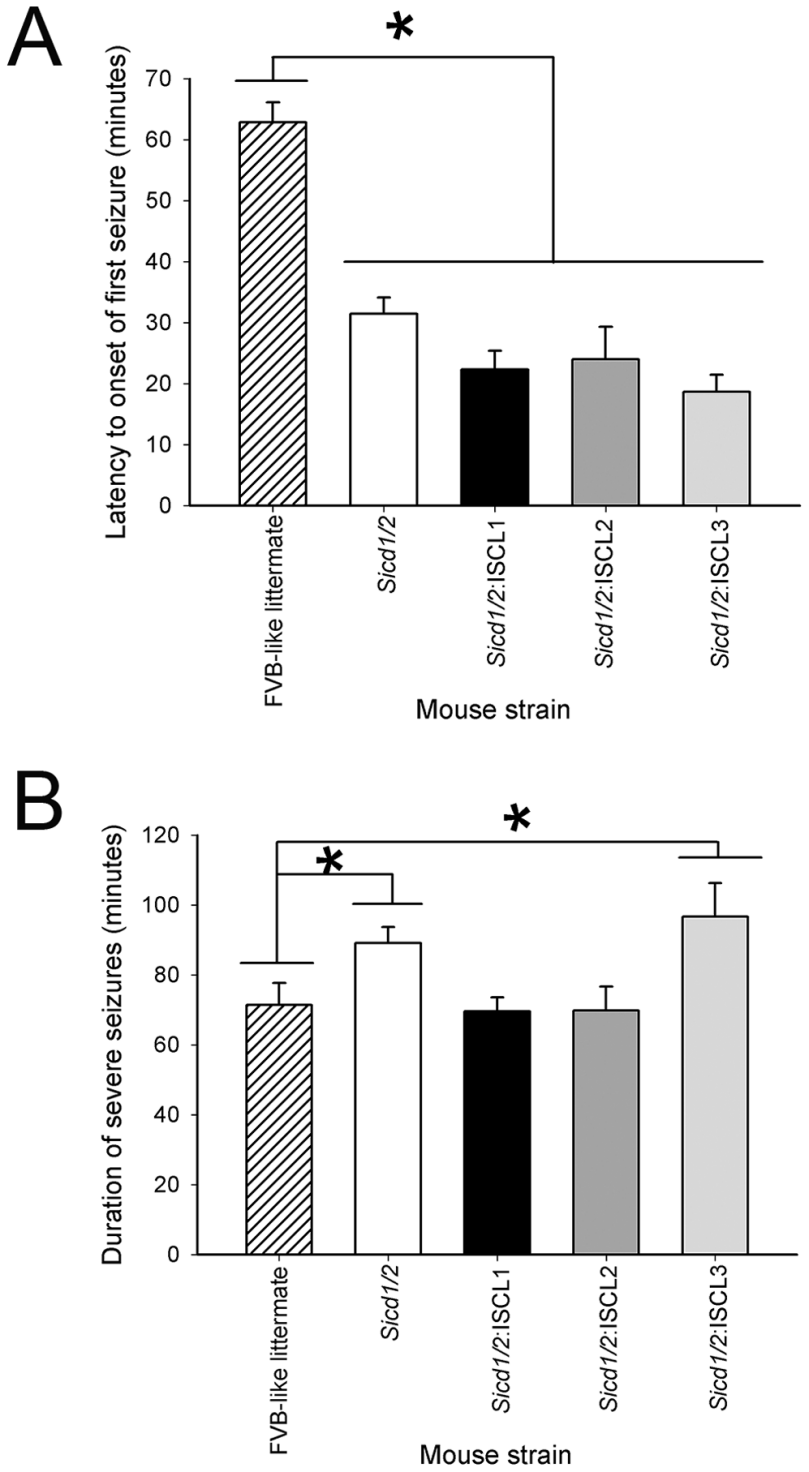
Histograms of seizure parameters in FVB.B6-*Sicd1/Sicd2* double congenic, and FVB.B6-*Sicd1/Sicd2*-ISCL1-ISCL-3 double sub-congenic mice following KA-induced status epilepticus. A. Data represent latency scores in minutes (mean ± SEM) for FVB.B6-*Sicd1/Sicd2* (n = 30), FVB.B6-*Sicd1/Sicd2*-ISCL1 (n = 14), FVB.B6-*Sicd1/Sicd2*-ISCL2 (n = 7), FVB.B6-*Sicd1/Sicd2*-ISCL3 (n = 14), and FVB-like littermate (n = 14) strains. ANOVA testing showed a significant reduction in latency (F_4,78_ = 26.39; P<0.001) between all congenic strains and FVB-like littermates. B. Data represent the seizure duration scores in minutes (mean ± SEM) for FVB.B6-*Sicd1/Sicd2*, FVB.B6-*Sicd1/Sicd2*-ISCL1, FVB.B6-*Sicd1/Sicd2*-ISCL2, FVB.B6-*Sicd1/Sicd2*-ISCL3, and FVB-like littermate strains. ANOVA testing showed a significant increase in duration of severe seizures between FVB.B6-*Sicd1/Sicd2* and FVB-like littermates (F_1,43_ = 4.87; P = 0.03) and between FVB.B6-*Sicd1/Sicd2*-ISCL3 and FVB-like littermates (F_1,27_ = 4.93; P = 0.04).

Using these three interval-specific double congenic lines (ISCL), we assessed progeny for differences in latency to seizure onset and duration of severe seizures following systemic administration of kainic acid. As shown in Figure 5, the latency to the onset of the first severe seizure differed significantly among all 3 ISCL double congenic strains and FVB-like littermates (F_4,78_ = 26.39; P<0.001). In particular, a reduction of nearly 60% was observed amongst all three ISCL strains, as compared to the parental FVB strain. In contrast, seizure duration was only significantly increased in *Sicd1/Sicd2*-ISCL3 as compared to the parental FVB strain (F_1,27_ = 4.93; P = 0.04). Seizure duration in *Sicd1/Sicd2*-ISCL1 and *Sicd1/Sicd2*-ISCL2 was not different from that observed in the parental FVB strain.

### Susceptibility to seizure-induced cell death in interval-specific double congenic lines

**Figure 6:**
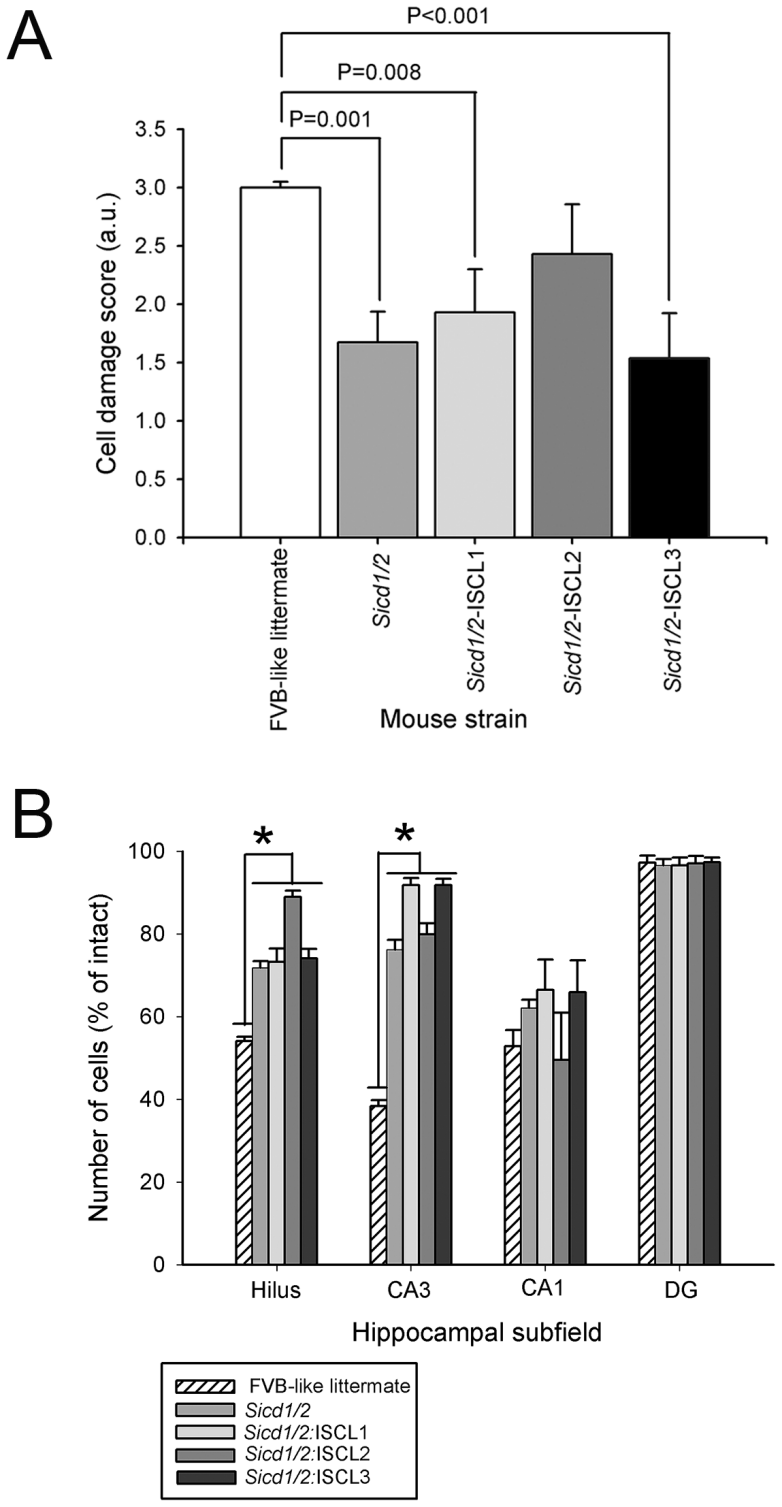
Kainate-induced cell death susceptibility phenotype of FVB.B6-*Sicd1/Sicd2* double congenic, and 3 FVB.B6-*Sicd1/Sicd2* double sub-congenic strains. Three *Sicd1/Sicd2* double sub-congenic lines (ISCLs1-3) were developed and tested to attain higher resolution mapping of the *Sicd1/Sicd2* double congenic strain. Strains were produced as described in Materials and Methods. A. Data represent neuronal damage scores for the entire right and left hippocampus [in arbitrary units (a.u.), mean ± standard error of the mean (SEM)] for FVB.B6-*Sicd1/Sicd2*, FVB.B6-*Sicd1/Sicd2*-ISCL1, FVB.B6-*Sicd1/Sicd2*-ISCL2, FVB.B6-*Sicd1/Sicd2*-ISCL3, and FVB-like littermates. The extent of neuronal damage was indexed with a histological damage score. A statistically significant difference in cell loss throughout the hippocampus was observed in FVB.B6-*Sicd1/Sicd2* double congenics (F_1,43_ = 11.75, P = 0.001), FVB.B6-*Sicd1/Sicd2*-ISCL1 (F1,27 = 8.38, P = 0.008), and FVB.B6-*Sicd1/Sicd2*-ISCL3 (F1,27 = 14.33, P<0.001) at 7 days following KA administration as compared to FVB-like littermates. B. Quantitative analysis of neuronal density in hippocampal subfields following KA administration to FVB.B6-*Sicd1/Sicd2*, FVB.B6-*Sicd1/Sicd2*-ISCL1, FVB.B6-*Sicd1/Sicd2*-ISCL2, FVB.B6-*Sicd1/Sicd2*-ISCL3, and FVB-like littermate mice. Strain-dependent differences in cell loss in the dentate hilus (F_4,69_ = 11.15; P<0.001) and area CA3 (F_4,69_ = 32.95; P<0.001) were observed at 7 days following KA administration. Data represent the mean ± SEM of at least 7 mice/strain. CA1 and CA3, hippocampal subfields; DG, dentate gyrus.

As shown in Figure 6A, nearly all double congenic interval-specific lines showed reduced excitotoxic cell death throughout the hippocampus similar to the FVB.B6-*Sicd1/Sicd2* double congenic strain, as compared to the FVB-like littermates. FVB.B6-*Sicd1/Sicd2*-ISCL1 (F_1,27_ = 8.38; P = 0.008) and –ISCL3 (F_1,27_ = 14.33, P<0.001) exhibited significantly reduced neuronal damage scores (1.93±0.37 and 1.54±0.39, respectively) as compared to their FVB-like littermates (3.00±0.05). However, when cell damage was assessed across hippocampal subfields, all double sub-congenic lines showed significant protection against hippocampal cell loss in the dentate hilus and in area CA3 seven days following kainate administration than their respective FVB-like littermates (Figure 6B). Quantitative analysis of hippocampal subfield group means revealed that while all sub-congenics showed statistically significant reduced cell loss in the dentate hilus as compared to FVB-like littermates (F_4,69_ = 11.15; P<0.001), *Sicd1/Sicd2*-ISCL2 showed significantly less cell death as compared to any of the other congenic strains. In particular, *Sicd1/Sicd2*-ISCL2 lost on average only 12% hilar neurons as compared to the approximately 27% lost by *Sicd1/Sicd2* or *Sicd1/Sicd2*-ISCL1 or –ISCL3 strains. Similarly, all interval-specific double congenic lines showed significant protection against CA3 cell loss as compared to their FVB-like littermates (Figure 6B). Within area CA3, whereas all ISCLs showed a reduction in the extent of cell loss as compared with FVB-like littermates, ISCL-1 and ISCL-3 showed the greatest protective effect with only a 9% cell loss. No congenic strains showed any statistically significant protection against CA1 cell loss

## Discussion

Susceptibility to seizure-induced excitotoxic cell death is under complex genetic control and results from the interaction between multiple host genes. The host genetic architecture responsible for modulating susceptibility to seizure-induced excitotoxic cell death was previously investigated in our laboratory by performing a linkage analysis on N2 animals from a backcross between FVB/NJ (FVB) and C57BL/6J (B6) mice. Linkage analysis led to the mapping of three QTLs (*Sicd1*, *Sicd2* and *Sicd3*) located on chromosomes (Chrs) 18, 15, and 4, respectively, linked to susceptibility to seizure-induced excitotoxic cell death. Multilocus models demonstrated that the 3 QTLs could explain the difference in susceptibility and that the three QTLs together account for nearly 25% of the trait variance for both genders combined [30]. We previously examined all two-way epistatic interactions and detected three significant epistatic interactions, one between chromosome 15 and chromosome 18. The Chr 15 locus (*Sicd2*) contributes to kainate-induced cell death susceptibility, not only as a main effector, but also as an interacting QTL [30]. Thus, we generated a double congenic strain, FVB.B6-*Sicd1/Sicd2*, where we introgressed the interval containing the putative resistant B6 alleles on Chr 18 (*Sicd1*) and Chr 15 (*Sicd2*) onto the susceptible FVB background, to determine whether these two QTLs (*Sicd1* and *Sicd2*) affect the phenotype of seizure-induced cell death in an additive manner.

Phenotypic analysis of the extent of seizure-induced cell death in the FVB.B6-*Sicd1/Sicd2* double congenic strain confirmed that both loci play an intrinsic role in susceptibility to seizure-induced cell death. This conclusion is supported by data demonstrating the dramatic reduction in susceptibility to seizure-induced cell death within the FVB.B6-*Sicd1/Sicd2* strain relative to the FVB-like littermates. Indeed, we did observe a significant decrease in seizure-induced cell death in the double congenic strain as compared to FVB.B6-*Sicd1*, FVB.B6-*Sicd2*, or FVB-like littermates. We observed an epistatic effect in this study, namely, the summation effect of two *Sicd* resistant regions, *Sicd1* (Chr 18) and *Sicd2* (Chr 15), each of which decreases the extent of seizure-induced excitotoxic cell death in the hippocampus on its own, and demonstrated that these two loci can modulate susceptibility to seizure-induced cell death in a synergic manner. Thus, introgressing both B6 *Sicd1* and *Sicd2* QTL-containing regions into the same FVB genetic background allowed confirmation of the epistatic interaction observed in our prior genome scan [30] and demonstrated the interactive effect between the two loci. Results of this study underline the importance of considering epistatic QTL mapping in complex traits. Epistatic QTL-mapping studies in model organisms have detected many new interactions and established that epistasis explains a large proportion of the genetic regulation of complex traits [57].

As previous studies have suggested that reduced susceptibility to seizure-induced cell death could partially result from a reduction in seizure activity [21], [58]–[60], we examined several seizure parameters, seizure latency and seizure duration, in homozygous FVB.B6-*Sicd1/Sicd2* double congenic mice. Interestingly, although FVB.B6-*Sicd1/Sicd2* double congenic mice showed reduced susceptibility to seizure-induced cell death, they exhibited a significant decrease in the latency to the onset of the first seizure and a significant increase in seizure duration. Worthy of note is the fact that decreased seizure latency and increased seizure duration usually imply increased seizure sensitivity, and, as a consequence, increased cell death. However, we found a considerable discrepancy within our double congenic strain. Although we would have expected increased cell death, due to the decreased seizure latency and increased seizure duration, we instead found increased protection against seizure-induced cell death. These results are similar to what we observed in earlier studies with the FVB.B6-*Sicd2* single congenic mice [33], and suggest that FVB.B6-*Sicd1/Sicd2* mice may actually have enhanced kainate seizure sensitivity. Our observation of different and apparently contrasting effects between ictogenesis and susceptibility to seizure-induced cell death has been reported in a number of other studies using the kainate or pilocarpine status epilepticus paradigms [25], [26], [29], [31]. These studies have reported that seizure severity does not necessarily correlate with excitotoxic neuronal loss and that the duration or severity of seizure activity is not necessarily predictive of subsequent cell death [26], [29]. As well, a number of studies have indicated that there are substantial differences in seizure susceptibility induced by electrical or chemical means in different strains that are genetically determined [21], [25], [61]–[64]. In particular, it has been reported that seizure susceptibility is a polygenetic phenomenon with loci of significant effect on chromosomes 1, 5, 7 and 15 [61], [63], [65]–[66]. Together, this suggests that a discrepancy between seizure sensitivity and seizure-induced cell death is likely due to activation of different mechanisms and/or different genes related to increased excitability versus those that protect neurons. Our results suggest that the B6 introgressed regions on Chrs 18 and 15 not only influence susceptibility to seizure-induced cell death but may also influence particular aspects related to seizure sensitivity (e.g. seizure latency and seizure duration). Thus, although *Sicd1/Sicd2* exerts definitive protective effects on the development of susceptibility to seizure-induced cell death, these neuroprotective effects are not a result of reduced seizure sensitivity.

In the present study, we produced three double congenic ISCLs carrying differing intervals from Chr 18 or Chr 15 of B6 on an FVB-derived susceptible background. All of these sub-congenic strains had decreased susceptibility to seizure-induced cell death as compared with their FVB-like littermates when cell damage was assessed by regional analysis of hippocampal subfields, owing to the effect of B6 alleles on Chr 18 and Chr 15. In particular, all strains were significantly more protected from seizure-induced excitotoxic cell death than either the *Sicd1* or *Sicd2* strains, suggesting that both loci are essential for the interlocus genetic interactions. A significant protective effect was observed in all three ISCL strains throughout area CA3 and the dentate hilus; a pattern reminiscent of what we had observed in FVB.B6-*Sicd1/Sicd2* double congenic mice. The most dramatic reduction in seizure-induced cell death susceptibility in area CA3 was expressed in FVB.B6-*Sicd1/Sicd2*-ISCL1 and –ISCL3, which carried reduced B6-derived regions on both Chr 18 (FVB.B6-*Sicd1/Sicd2*-ISCL3) and Chr 15 (FVB.B6-*Sicd1/Sicd2*-ISCL2) on an FVB-derived susceptible background. Both of these ISCLs showed a strong B6 protective effect on seizure-induced cell death in area CA3 as compared with FVB-like littermates, indicating that protective B6 alleles are contained in a minimal 2 cM introgressed region spanning the distal part of Chr 18 and in a 19.5 cM interval spanning the proximal part of Chr 15.

In contrast, FVB.B6-*Sicd1/Sicd2*-ISCL2 showed significantly reduced susceptibility to seizure-induced excitotoxic cell death in the dentate hilus, as compared with FVB-like littermates, the FVB.B6-*Sicd1/Sicd2* double congenic strain, as well as all other ISCLs. This result suggests that the gene(s) responsible for reduced hilar susceptibility is located in the 2 cM interval on Chr 18 flanked by markers D18Mit129 and D18Mit25 and in the 29.5 cM interval on Chr 15 flanked by markers D15Mit174 and D15Mit156. Consequently, the reduction in cell death may be attributed to the B6 alleles from these two introgressed regions. Differences in the extent of cell death across hippocampal subfields could result from differences in gene composition within each interval and also from differences in seizure activity. Moreover, each of the FVB.B6-*Sicd1/Sicd2*-ISCL strains further narrows both QTL intervals to smaller regions of murine Chr 18 and Chr 15 and supports the proposition that the protective genes are contained within these smaller intervals.

We examined several seizure parameters in these three ISCLs, such as seizure latency and seizure duration. Despite the dramatic protective effect that we observed in ISCL3 with regard to susceptibility to seizure-induced cell death in area CA3 as well as in the dentate hilus, in comparison with the FVB-like littermates, we observed a significant reduction in latency to first seizure and a significant increase in seizure duration. The shortened latency and increased duration of seizures gives emphasis to the fact that ISCL-3 may actually have increased KA seizure sensitivity. Nevertheless, it is important to note that, although ISCL-3 shows enhanced protection against susceptibility to seizure-induced cell death within area CA3 and the dentate hilus, these protective effects are not the result of diminished seizure sensitivity. Furthermore, while we did see a dramatic reduction in the latency to the first seizure amongst all 3 ISCL strains, we did not see a significant difference in seizure duration in the ISCL1 and ISCL2 strains, as compared with FVB-like littermates. These results seem to suggest that, in contrast to the FVB.B6-*Sicd1/Sicd2* double congenic mice and the FVB.B6-*Sicd1/Sicd2*-ISCL3 mice, genes contained within the B6 interval of ISCL-1 and -2 likely have a primary role in modulating seizure-induced cell death rather than seizure sensitivity.

In conclusion, this study revealed an epistatic relationship between *Sicd1* and *Sicd2* that is consistent with the hypothesis generated from a whole-genome scan study [30]. It identified an epistatic effect, implying the *Sicd1* QTL on Chr 18 has a synergic and additive effect with the *Sicd2* QTL on Chr 15. Susceptibility to seizure-induced cell death is likely the result of multiple alleles interacting at multiple loci, as we found that two independently beneficial loci have their joint effect increased, resulting in enhanced protection against seizure-induced cell death. Our results indicate that reduced intervals of both of our QTLs (*Sicd1* and *Sicd2*) are major determinants of the difference in seizure-induced cell death susceptibility between B6 and FVB mice. Further studies on the action and interactions of these loci as well as the eventual identification of *Sicd* genes will enable us to address the relevance of their human counterparts, thus providing a clue for the potential differences in susceptibility to seizure-induced cell death observed in the human population.
